# The Role of Inflammation in Cognitive Impairment of Obstructive Sleep Apnea Syndrome

**DOI:** 10.3390/brainsci12101303

**Published:** 2022-09-27

**Authors:** Chunlan Yang, Yuanqing Zhou, Haijun Liu, Ping Xu

**Affiliations:** Department of Neurology, Affiliated Hospital of Zunyi Medical University, Zunyi 563000, China

**Keywords:** obstructive sleep apnea syndrome, cognitive impairment, inflammation, chronic intermittent hypoxia

## Abstract

Obstructive sleep apnea syndrome (OSAS) has become a major worldwide public health concern, given its global prevalence. It has clear links with multiple comorbidities and mortality. Cognitive impairment is one related comorbidity causing great pressure on individuals and society. The clinical manifestations of cognitive impairment in OSAS include decline in attention/vigilance, verbal–visual memory loss, visuospatial/structural ability impairment, and executive dysfunction. It has been proven that chronic intermittent hypoxia (CIH) may be a main cause of cognitive impairment in OSAS. Inflammation plays important roles in CIH-induced cognitive dysfunction. Furthermore, the nuclear factor kappa B and hypoxia-inducible factor 1 alpha pathways play significant roles in this inflammatory mechanism. Continuous positive airway pressure is an effective therapy for OSAS; however, its effect on cognitive impairment is suboptimal. Therefore, in this review, we address the role inflammation plays in the development of neuro-impairment in OSAS and the association between OSAS and cognitive impairment to provide an overview of its pathophysiology. We believe that furthering the understanding of the inflammatory mechanisms involved in OSAS-associated cognitive impairment could lead to the development of appropriate and effective therapy.

## 1. Introduction

OSAS involves the entire (apnea) or partial (hypopnea) collapse of the upper airway that leads to brief (tens of seconds) and repeated interruptions of breathing during sleep, which causes intermittent hypoxia (IH), hypercapnia, and arousal. The sleep architecture of patients with OSAS is altered, including issues such as sleep fragmentation, slow wave sleep duration decline, and non-rapid eye movement stages 1 and 2 increase [1,2]. The night-time symptoms of OSAS include snoring, breathing breaks, superabundant salivation, excessive sweating, gastroesophageal reflux, nocturia, and headache. OSAS is diagnosed using the apnea hypopnea index (AHI) and polysomnography, which are both gold standard diagnostic tools. The AHI refers to the number of apnea or hypopnea episodes per hour occurring during sleep. It has been reported that obesity, age, and sex may be the three most significant risk factors for OSAS [3]. The prevalence of OSAS in the adult population has been reported to range from 9% to 38% (men, 13–33%; women, 6–19%) [4]. With increasing age, the prevalence, related comorbidities, and phenotypic presentation can vary widely [5]. Some studies have reported a prevalence of >50% in older adults [4]. OSAS has a role in hypertension, type 2 diabetes mellitus, and cognitive decline [6,7,8]. In patients with OSAS, inflammatory cytokines such as tumor necrosis factor (TNF)-α and interleukin (IL)-6, induced by the nuclear factor kappa B (NF-κB) pathway, have been shown to increase [9]. Both NF-κB and hypoxia-inducible factor 1 alpha (HIF-1α) are crucial transcription factors that participate in inflammation and hypoxic diseases, respectively [10]. Neuroinflammation plays a crucial role in cognitive impairment and memory deficits [11].

Cognitive dysfunction is a syndrome involving cognitive decline with or without functional impairment, with a risk of progression towards dementia. Cognitive impairment is an important clinical manifestation of OSAS involving attention/vigilance decline, verbal–visual memory decrease, visuospatial/structural ability impairment, and executive dysfunction [12]. It is reported that OSAS injures the hippocampus, related to learning and memory, which negatively influences the patient quality of life and increases the risk of work- and traffic-related accidents [13,14,15]. IH, sleep fragmentation, neuroinflammation, and cerebrovascular changes may be mechanisms of cognitive impairment related to sleep apnea [16,17]. It has been reported that sleep fragmentation is related to attention and executive function impairment [18]. The apnea trends during REM sleep in OSAS can cause disruption to the memory consolidation processes [19]. Continuous positive airway pressure (CPAP) is currently one of the most effective therapies for OSAS; it can partially repair injury to the hippocampus and improve related functional deficits [20,21].

Given the harm that cognitive impairment in OSAS can cause and its high prevalence, developing an effective treatment is of high concern. In this review, we examine the relationship between OSAS and cognitive impairment, especially from the perspective of inflammation, hoping to provide a basis for future studies and the development of appropriate therapy. IH is considered the major risk factor for morbidity and mortality in OSAS, and we mainly focus on this subject.

### 1.1. Cognitive Impairment in OSAS

Cognition processes are completed in different regions of the brain, with investigations mainly focusing on the frontal cortex and hippocampus [22]. The hippocampus is an essential area participating in neurogenesis and dentate gyrus (DG) function and hippocampal circuitry, as well as participating in the processes of learning and memory (sensory, short-term, and long-term memory) [23]. The hippocampus can be damaged because of hypoxia, oxidative stress, and inflammation, which are all considered pathological manifestations of OSAS [24]. Normally, the cerebral autoregulation mechanism can preserve cerebral perfusion despite changes in blood pressure [25]. However, nocturnal intracranial hemodynamics and oxygen saturation levels change in OSAS [25]. During obstructive apnea, cerebral blood flow velocity gradually increases and then sharply declines below the baseline, which may lead to nocturnal cerebral ischemia [25].

In the cortex and brainstem of animals, intermittent hypoxia can cause neuronal degeneration and axonal dysfunction [26]. Rats exposed to IH have been found to have learning disorders [27]. IH causes hypomyelination and decline in myelin-associated protein expression in the cerebral cortex [28,29]. It has been reported that IH preferentially activates the inflammatory pathways that are mediated by NF-κB, which is a key transcriptional activator of HIF-1α and is essential for HIF-1α deposition during hypoxia [30,31]. HIF-1α causes the translocation of NF-κB to the nucleus, promoting IL-1β and TNF-α expression among other pro-inflammatory cytokines [32].

There are two types of IH, namely, acute IH and chronic IH (CIH), the former usually occurring for several minutes to hours and the latter occurring for several days, weeks, or years. Compared with continuous hypoxia, IH in OSAS presents with higher frequency and more severe hypoxia, as well as greater change in blood oxygen saturation levels [33]. CIH drives neuronal apoptosis in the hippocampal CA1 region, contributing to cognitive dysfunction, as the CA1 region is most susceptible to hypoxic injury [34,35]. The G protein in the DG may be inactivated because of hypoxia, resulting in alterations in hippocampal function [36]. Further, OSAS-related hypoxemia increases free radicals and inflammation and likely harms neurons in several brain regions, resulting in the destruction of endothelial and neuronal integrity [37].

CIH promotes the deposition of reactive oxygen species (ROS), causing disorders of the mitochondria and endoplasmic reticulum that can then result in a reduction in adenosine triphosphate production, declining antioxidant capacity, protein overproduction, DNA oxidation, lipid peroxidation, and impairment of cells and tissues [38]. CIH resembles the ischemia–reperfusion process, maintaining a cycle of hypoxia and reoxygenation, with repetitive episodes of hypoxia increasing ROS production [39,40]. Oxidative stress increases the inflammatory response, with inflammation then strengthening oxidative stress [33]. The accumulation of oxidative stress products in CIH damages neurons and neural signaling pathways, which may play a significant role in the development of cognitive decline in OSAS [41]. Furthermore, the nitric oxide pathway disorder in OSAS is likely to disturb neurons, synapses, and neurotransmission, resulting in synaptic loss and neuronal damage [42,43].

**Figure 1:**
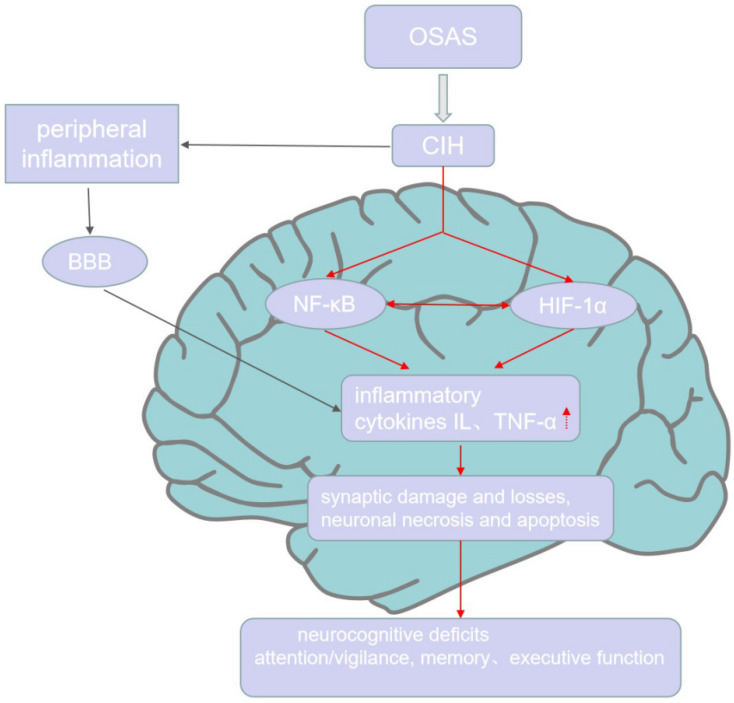
Inflammation and cognitive impairment in OSAS. CIH, the characteristic of OSAS, causes peripheral inflammation and access the CNS through BBB to induce the production of NF-κB and HIF-1α, which both promote the expression of inflammatory cytokines in the CNS. Further, HIF-1α and NF-κB can interact with each other. The high level of inflammation in CNS further causes neuronal necrosis, apoptosis, synaptic damage, and losses, which finally leads to neurocognitive deficits, including attention/vigilance decline, verbal–visual memory decrease, and executive dysfunction.

It is suggested that OSAS is a low-grade chronic inflammatory disease [44]. Circulating markers of inflammation such as C-reactive protein (CRP), cytokines, and adhesion molecules have been observed [45,46]. CRP levels are positively related to AHI, the arousal index, and oxygen saturation levels [47,48]. The severity of nocturnal hypoxemia affects cytokine and adhesion molecule levels [49]. Numerous studies have shown that peripheral inflammation and changes to the gut microbiome can enhance neuroinflammation and promote neurodegeneration [50,51,52]. Hypoxia and oxidative stress in OSAS cause increases in neuroinflammatory cytokines and cellular dysfunction, leading to chronic damage and neuronal cell apoptosis, which causes cognitive dysfunction [41] (Figure 1).

### 1.2. Inflammation in Cognitive Impairment

A strong association between chronic inflammation and age-related pathologies such as cardiovascular diseases, diabetes mellitus, and dementia has been reported [53]. Chronic systemic inflammation is considered one of the pathological mechanisms in neurodegenerative diseases such as Alzheimer’s disease (AD) [54]. Neuroinflammation involves numerous pathways, and cytokines may participate in AD pathology [55]. When the brain’s immune cells are activated, chronic inflammation and brain damage are invoked [56]. Viral infection can also damage the nervous system, which may be mediated through an immune reaction [57]. For example, the coronavirus disease, or COVID-19, which is induced by severe acute respiratory syndrome coronavirus 2, may lead to cognitive decline and neurodegeneration because of its common clinical presentation, that is, acute respiratory distress syndrome [58,59].

Inflammatory responses significantly reduce nerve conduction speed, axonal excitability, and synaptic efficiency, and negatively affect the rate of signal transduction and neuronal integrity [60,61,62]. An inflammatory response generally involves the coordinated activation of numerous signaling pathways that adjust pro- and anti-inflammatory expression in resident tissue cells and leucocytes [63]. The persisting imbalance between pro- and anti-inflammatory cytokines injures neuronal integrity [64]. These cytokines are mostly produced by Th cells, peripheral antigen-presenting cells, and macrophages, triggering the central and peripheral inflammation cascade in pathological conditions [65], and they are considered potential hallmarks of dementia and memory deficiency [66].

In pathological conditions, the lasting activation of microglia induces a chronic inflammatory process, with an increase in pro-inflammatory cytokines and a decline in neuroprotective factors, leading to neurodegeneration [67]. In a mouse model, TNF-a and IL-1β restrained long-term potentiation, which is a pattern of synaptic activity basic for memory [68]. IL-1β participates in the release of tryptophan metabolites as well as oxidative stress, leading to delayed neurogenesis and fatigue [69]. In NLRP3 knockout mice, microglial activation induced by CIH decreased, concomitantly with the reduction of oxidative stress levels [70]. NLRP3 deficiency can protect against CIH-induced neuroinflammation by promoting Parkin-dependent mitophagy [70]. The NLRP3 inflammasome contributes to several inflammatory disorders, such as AD, diabetes, and atherosclerosis [71]. NLRP3 is a type of cytosolic multiprotein complex related to the innate immune response. Furthermore, significant differences in NLR were found in a progressive supranuclear palsy Richardson syndrome group compared with a control group, which suggested that the neutrophil-to-lymphocyte ratio (NLR) may be a non-specific parameter in neurodegenerative diseases. The NLR and platelet-to-lymphocyte ratio increase in alpha-synucleinopathies and Parkinson’s disease (PD) [72,73]. In the NLR inflammasome family, NLRP3 is highly expressed in microglia, whereas NLRP1 and NLRP2 are more highly expressed in neurons and astrocytes, respectively [74]. Inflammasome pharmacological blockage may be a potential treatment for OSAS-related cognitive impairment.

The primary aim of inflammation is to protect neural integrity in the central nervous system (CNS), but chronic inflammation has a pernicious effect and further enhances neuronal injury [64]. For instance, in diabetes mellitus, decreases in TNF-α and IL-1β may ameliorate related cognitive decline [11]. Chronic inflammation can disturb glutamate neurotransmitters, leading to neurocircuitry malfunction in the glia [75]. As the principal pathophysiological process of OSAS, CIH promotes neuroinflammation and oxidative stress, causing OSAS-related cognitive impairment [70].

### 1.3. The Inflammation in Cognitive Impairment of OSAS

It is known that neuroinflammation contributes to CIH-induced nerve cell damage, and inflammatory cytokines increase in the hippocampus of rats with CIH [76]. The mechanisms of chronic inflammation leading to cognitive impairment may be summarized as follows: (1) cytokines, which regulate gene expression of growth factors critical to synaptic plasticity and memory [77]; (2) durative activation of microglia, leading to neuronal damage [78]; and (3) inflammation influencing neuronal morphology, especially in terms of reorganizing neuronal dendritic spines in vulnerable regions [77].

Neuroinflammation and blood–brain barrier (BBB) hyperpermeability are possible mechanisms in OSAS contributing to cognitive impairment [25]. The BBB is a physical barrier comprising endothelial cells, astrocytes, and pericytes, which works to keep damaged molecules away from the CNS [79]. In mice, IH invoked low-grade neuroinflammation in the dorsal hippocampus, including early cytokine and delayed microglial changes, which are all related to IH-induced cognitive impairment [80]. *Lycium barbarum* polysaccharides can promote hippocampal neurogenesis and alleviate the apoptotic signaling cascades induced by oxidative stress and inflammation to improve CIH-induced hippocampal-dependent spatial memory deficits [81]. Sesamol possesses antioxidant, anti-inflammatory, and neuroprotective capacities [39]. In CIH-exposed rats, sesamol alleviated learning and memory impairment and reduced TNF-α and IL-1β levels in the hippocampus [39]. As a central participant in adjusting the immunological response to infection and inflammation, NF-κB is involved in the HIF-1α mRNA transcriptional response and in the downstream signaling of Toll-like receptors (TLRs) [82]. IH promotes the activation of NF-kB and other inflammation-related transcription factors in monocytes and neutrophils [83,84,85].

#### 1.3.1. NF-κB: The Probable Connection between OSAS and Neurocognitive Impairment

In the CNS, numerous neurotrophic factors, cytokines, and neurotransmitters can activate NF-κB, which can regulate neuronal survival and death, myelination of peripheral nerves, and synaptic function [86]. For instance, the brain-derived neurotrophic factor (BDNF) contributes to memory, learning, and behavior [87]. In developing peripheral and central neurons, NF-κB also positively regulates axonal and dendritic growth [88]. NF-κB is a typical pro-inflammatory signaling molecule [63]. It is a transcription factor system, and its homodimers or heterodimers of five structure-associated proteins comprise p65, RelB, p50 and p52, and c-Rel [88]. NF-κB dimers maintain an inactive form in the cytoplasm because the NF-κB-inhibitor (IκB) family, including Bcl-3, p100, p105, IκBα, IκBβ, IκBε, and IκBγ, combine with them [88]. Among these, the p65/p50 heterodimer is the most extensively expressed form, and the IκBα protein is the major inhibitor [88]. There are at least two independent pathways that activate the NF-κB pathway, namely, the canonical and the alternative [63]. These are distinguished by the differential requirement for IkappaB kinase (IKK) subunits [63]. The IKK complex includes a regulatory subunit IKKγ and two kinase subunits, IKKα and IKKβ [63]. With the help of the IKKγ subunit, IKKβ activates the canonical pathway through phosphorylating IκBs [89]. IKKα activates the alternative pathway through phosphorylating and processing the p52 precursor, p100 [90]. After NF-κB is activated, IκB is degraded in a proteasome-dependent manner [91].

**Figure 2:**
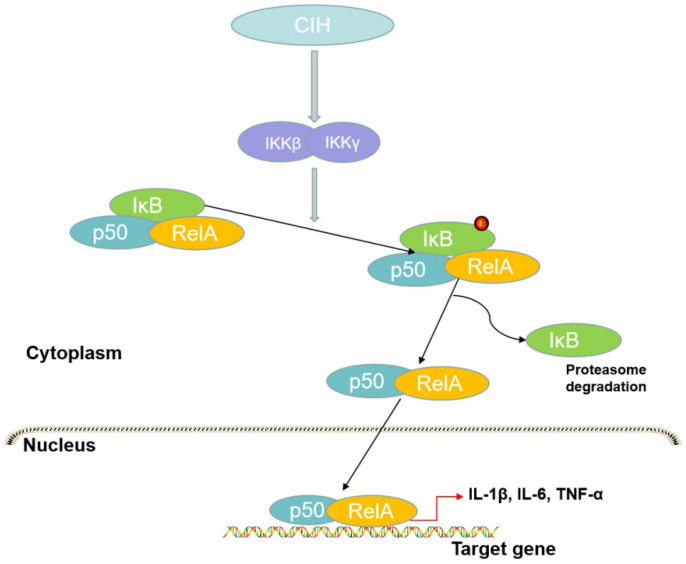
The canonical pathway of NF-κB. In this process, IKKβ activated by CIH leads to the phosphorylation of IκB to form the RelA/p50 complex. The complex translocates to the nucleus to promote the transcription of target genes and IκB is subsequently degraded by the proteasome.

The canonical pathway is essential in the mechanism of inflammation and plays a crucial role in innate immunity, whereas the alternative pathway participates in lymphoid organ development and adaptive immunity [92]. The canonical pathway activates the p65/p50 heterodimer [93] (Figure 2). In the canonical pathway, stimuli such as lipopolysaccharides (LPSs), interferon-gamma, and TNF-α promote the phosphorylation of IKK, participating in IκBα serine phosphorylation and degradation and influencing the release of NF-κB [94]. Pathogen-associated molecular patterns (PAMPs) and damage-associated molecular patterns (DAMPs) activate the immune system via pattern recognition receptors (PRRs) [95]. To recognize these signals, TLRs or inflammasomes mediate specific immune signaling pathways and then promote the subsequent activation of NF-κB [95].

TLR4 may also participate in OSAS, as monocytes in patients with OSAS express higher TLR4 at their surface [96]. Under hypoxic conditions, the signaling transduction of TLRs and related expression both increase, leading to activation of the NF-κB pathway [97]. When LPS binds to TLR4, the downstream cascade activates the pro-inflammatory NF-κB pathway, resulting in an increase in several pro-inflammatory molecules [98]. The NF-κB pathway participates in pathological brain inflammation and is associated with neuronal apoptosis, which may result in impaired cognitive function, as activated NF-κB can induce cytotoxic products that promote inflammation, oxidative stress, and apoptosis [99,100,101]. NF-κB activation is associated with the pathophysiology of OSAS, as it is activated in hypoxic conditions, while IH is a strong pro-inflammatory stimulus [30,85].

In the first few hours of chronic hypoxia, NF-κB levels increased, and NF-κB activation in hypoxia is mediated by the PI3K/PKB signal pathway [102,103]. This pathway is induced either by ROS production or by membrane receptors [103]. Protein kinase D2 is another NF-κB activation pathway in chronic hypoxia [104]. Further, p38 mitogen-activated protein kinase is important in the IH-induced process of NF-κB activation [105]. Many studies have shown that NF-κB and pro-inflammatory cytokines significantly contribute to neuronal dysfunction, and NF-κB, the pro-inflammatory transcription factor induced by IH, evokes OSAS systemic inflammation [30,85,106,107]. NF-κB activation would lead to endothelial dysfunction in endothelial cells obtained from patients with OSAS; however, this dysfunction could improve with effective CPAP therapy [108]. NF-κB plays a key role in the immune and inflammatory responses and can also regulate the HIF-1α mRNA transcriptional response [82].

#### 1.3.2. HIF: A Factor That May Link OSAS with Neurocognitive Impairment

The active HIF transcriptional complex has three isoforms, namely, HIF-1, HIF-2, and HIF-3, which are composed of subunits HIF-α, HIF-β, and coactivator p300/CREB binding protein (CBP) [109]. The HIF transcriptional complex regulates the expression of multiple genes that enable a cell to resist a hypoxic environment [110].

There are three subtypes of HIF-α, HIF-1α, -2α, and-3α in humans, among which HIF-1α and HIF-2α are the most representative, whereas the role of HIF-3α has not been clearly elucidated [109]. Where HIF-1α expression is very wide, HIF-2α expression is tissue-specific [109]. HIF-1α can regulate gene expression to participate in vascular resistance, glucose metabolism, erythropoiesis/iron metabolism, and the circadian rhythm, with significant effects on various physiological and pathological processes [111]. In chronic hypobaric hypoxia, neuronal apoptosis induced by HIF-1α in the hippocampus is a significant cause of cognitive impairment [112]. During cerebral ischemic insults, HIF-1α has many neuroprotective effects [113,114]. The neuroprotective functions of HIF-1α may be mediated by proteins that are encoded by their target genes, such as erythropoietin, vascular endothelial growth factor, and the glucose transporter [115]. Therefore, HIF-1α has been considered a therapeutic target for cerebral ischemia [115].

Transcription factors and coactivators are transcriptional modulators of HIF-1α, with the former including NF-κB, signal transducers, and the signal transducer and activator of transcription 3, the latter including p300/CBP [116,117]. One study of HeLa cells showed that HIF-1α levels rose significantly when O_2_ concentrations fell to 6% (42 mmHg), with half-maxima at O_2_ 1.5% (10.5 mmHg) and maxima at O_2_ 0.5% (3.5 mmHg), but that HIF-1α rapidly declined when O_2_ concentrations were as high as 20% (140 mmHg), with a half-life < 5 min [118,119]. Normally, O_2_ induces the degradation of HIF-1α by prolyl hydroxylases (PHDs) [109]. Under chronic hypoxia, degradation is affected in that HIF-1α subunits access the nucleus to engage with the transcriptionally active HIF complex [109].

**Figure 3:**
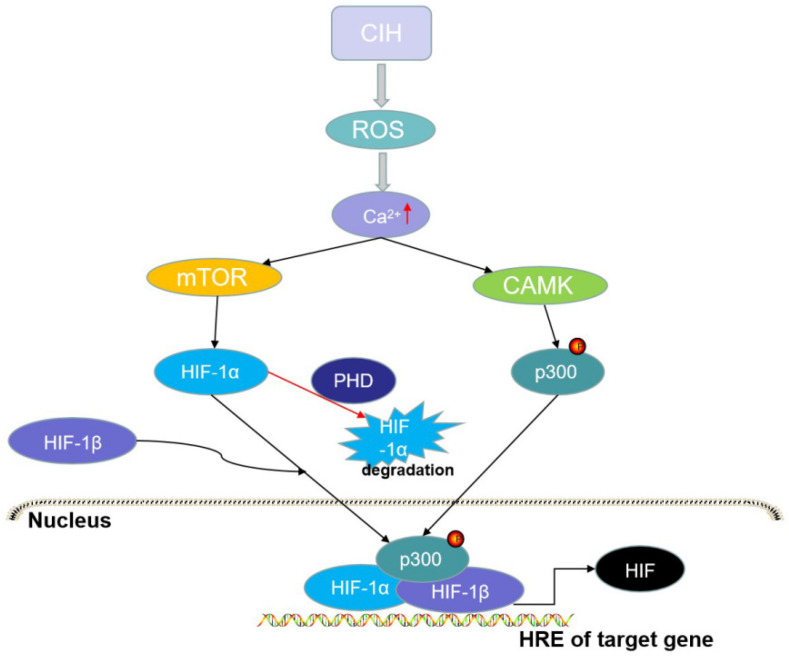
The activation of hypoxia-inducible factor (HIF)-1α. CIH can evoke oxidative stress and reactive oxygen species (ROS), while ROS can activate the Ca^2+^-dependent calpain proteases (CAMK) that can directly phosphorylate p300, and activate the mammalian target of rapamycin (mTOR) that promotes the expression of HIF-1α protein. The HIF-1α/HIF-1β/p300 complex in the nucleus can promote the transcription of HIF-1 genes. However, oxygen can induce the degradation of HIF-1α through prolyl hydroxylases (PHDs).

HIF-1α protein levels increase in hypoxia and decrease following reoxygenation [120]. Hypoxia may regulate the innate immune response under conditions of infection or inflammation by producing HIF-1α to regulate TLR expression and function [97,121]. Key inflammatory cytokines such as TNF-α can promote HIF-1α expression in innate immune cells [122]. IH is known to be key to myeloid dendritic cell (DC) function, and HIF-1α and HIF-2α are crucial transcription factors that regulate DC adaptation [123]. In in vivo studies, DCs lacking HIF-1α were shown to be the best inducers of the inflammatory response; therefore, IH evokes the selective upregulation of HIF-1α rather than of HIF-2α [124,125]. The ROS in CIH raises the intracellular calcium levels, resulting in protein kinase C-dependent mediation of the activity of the mammalian target of rapamycin to increase HIF-1α expression, which promotes the NOX_2_ gene expression responsible for the prooxidant enzyme, NADPH oxidase [126,127,128] (Figure 3).

In neurons, amyloid beta (Aβ) aggregation can activate some transcription factors, including HIF-1 and NF-κB, to promote the work of their downstream genes [129]. Hippocampal HIF-1α contributed to the increase in isoflurane-induced cognitive disorders [130]. Hypoxia promotes lactic acid and free radical generation, resulting in neuronal damage to the cerebellum and hippocampus [131]. Brain hypoxia influences the sporadic form of AD due to HIF-1 [132]. In hypoxemia, hypercapnia can damage the BBB by promoting HIF-1α nuclear translocation and the expression of AQP-4 and MMP-9 [133]. Furthermore, in hypoxia-activated astrocytes, hypercapnia induces HIF-1α nuclear translocation, and this may be an available target for improving cognitive impairment [133].

### 1.4. Impact of OSAS on Neuropsychological Diseases

Because of the negative effects of OSAS on the cardiovascular, neurocognitive, and metabolic states, attention to OSAS diagnosis and therapy is increasing [134]. OSAS is recognized as a risk factor for some neuropsychological diseases, such as AD, depression, and Parkinson’s disease (PD).

OSAS can raise the phosphorylation of tau proteins, increase the production of β-amyloid 42, and cause synaptic dysfunction, which are common pathophysiological changes in AD and OSAS [135]. In a rat OSAS model with CIH, IH significantly increased Aβ in the cerebrum and hippocampus of AD-transgenic mice [136,137]. It is suggested that hypoxia promotes tau hyperphosphorylation [138,139]. OSAS and AD are both low-grade inflammation diseases. In AD, the amyloid precursor protein and Aβ can activate microglia that in turn activate astrocytes, finally leading to the inflammatory response [140]. Furthermore, Aβ and tau initiate sterile inflammation and NLRP3 inflammasome signaling in AD [141]. NLRP3 inflammasomes lead to chronic neuroinflammation, neuronal death, and pyroptosis [142,143]. As mentioned above, IH invokes low-grade neuroinflammation in the hippocampus and causes memory deficits. Inflammation may comprise crosstalk between OSAS and AD.

OSAS is often accompanied by psychological symptoms, especially depressive disorders. Depression is a serious disorder; it afflicts more than 300 million people and is a major reason for disability [144]. Patients with major depressive disorder have issues with sleep, appetite, psychomotor activity, cognition, and mood. Patients with major depressive disorder often show thinking and memory impairment [145]. It has been suggested that inflammation contributes to depression. Peripheral inflammation causes major depressive disorder implicating the vagus nerve, leaky regions in the BBB, and cytokine transport systems [146,147]. However, the relationship between OSAS and depression is not clear. The night-time symptomatology of OSAS may be the main reason for depression in OSAS. More research is needed to explore the relationship between these two disorders.

PD, the second most common neurodegenerative disease after AD, mainly impacts the motor system and is associated with sleep and neuropsychiatric symptoms [148]. Insomnia, excessive daytime sleepiness (EDS), REM sleep behavior disorder, and sleep-disordered breathing are often present in neurodegenerative diseases [149]. Sixty percent of patients with PD show REM sleep behavior disorders [150]. The neuropathology of PD involves neuroinflammation in the substantia nigra pars compacta (SNc), progressive loss of dopaminergic neurons in the SNc, and Lewy bodies in different nuclei [151]. Continued microglial activation is essential to neuroinflammation and accelerates neurodegeneration in PD [152,153]. Furthermore, OSAS can promote PD onset, in which hypoxia may play an important role [154,155]. EDS is common between OSAS and PD. However, more research is needed to delineate the relationship between OSAS and PD.

### 1.5. The CPAP in Cognitive Impairment of OSAS

Nasal CPAP is the current treatment for moderate to severe OSAS because it splints the upper airway open; thus, offsetting negative suction pressure, leading to improvement in upper airway collapse [156,157]. CPAP maintains a set pressure during the respiratory cycle [158]. At the end of expiration, positive end-expiratory pressure (PEEP), which is the pressure in the alveoli, is higher than atmospheric pressure [159]. CPAP can maintain PEEP and reduce atelectasis, expand the surface area of the alveoli, ameliorate ventilation/perfusion matching, and improve oxygenation [159]. CPAP treatment for OSAS, especially if well adhered to, can ameliorate cognitive function and significantly reduce blood pressure and refractory hypertension [160]. Furthermore, CPAP treatment has been found to ameliorate cardiovascular and cerebrovascular responses, cognitive performance, and inflammation [161].

It has been reported that CPAP can produce small to moderate amelioration in executive function [162,163]. Further, memory may improve after CPAP treatment, although significant improvement appears to require >6 h of therapy [164]. Further, a longer treatment duration (>3 months) and adequate adherence to treatment (≥4 h/night) can effectively decrease systemic inflammation [165]. Elevated NF-κB and HIF-1α levels in OSAS decline after CPAP treatment [166]. Although CPAP remains the current gold standard therapy for OSAS, it is poorly tolerated by many patients, and its limited amelioration and its refractoriness in relation to neurological deficits have long been recognized [17,167]. One explanation for these findings may be poor adherence to treatment or irreclaimable brain injury from long-standing OSAS [164,168]. Furthermore, the CPAP effect on depressive symptoms is indefinite. Therefore, it is necessary to develop more effective and personalized interventions for patients with OSAS and neurocognitive impairment, depending on the OSAS sub-phenotype and its symptoms. Inflammation may be taken into account. For example, MiR-224-5p reduces microglial inflammation by decreasing NLRP3 expression and finally influences the NLRP3/IL-1β pathway in the hippocampus, which suggests that miR-224-5p may be a potential treatment target for OSAS [169]. In a mouse model, a TLR-4 receptor antagonist or blocking TNF-α to downregulate IL-1 could improve neuroinflammation and cognition [170,171]. Furthermore, surgery may be beneficial for the recovery of brain structures and functions by improving disease severity and systemic inflammation [172]. Therefore, NLRP3, NF-κB, and HIF may constitute treatment targets to improve cognition in OSAS, and this study’s focus on the role of inflammation in OSAS-related cognitive impairment is valuable. Therefore, NLRP3, NF-κB, and HIF may constitute treatment targets to improve cognition in OSAS, and this study’s focus on the role of inflammation in OSAS-related cognitive impairment is valuable.

## 2. Conclusions

Cognitive impairment and OSAS are major health concerns significantly affecting patients. The most pernicious feature of OSAS is CIH, which contributes to persistent, chronic inflammation and promotes the activation of NF-κB and HIF. Normally, inflammation is a beneficial process to eliminate harmful stimuli and repair damaged tissue. However, when a stimulus remains present for long, the inflammatory response can change into chronic inflammation and become deleterious. CPAP therapy remains the best treatment for OSAS, but its ability to ameliorate cognitive impairment is suboptimal. A more thorough investigation of the inflammatory mechanisms involved in OSAS-associated cognitive impairment is expected to have translational implications and provide a potential therapeutic target to add to or replace the current treatment. In addition, the potential of anti-inflammatory therapy remains to be elucidated, and further research is needed to this end. In the future, we expect a novel pharmacological agent to treat cognitive impairment and other OSAS-related comorbidities.

## Data Availability

It is not applicable to this article because no datasets were generated or analyzed during the review.

## Abbreviations

OSAS = obstructive sleep apnea syndrome; CIH = chronic intermittent hypoxia; NF-κB = nuclear factor kappa B; HIF = hypoxia-inducible factors; CPAP = continuous positive airway pressure; IH = intermittent hypoxia; SWS = slow wave sleep; NREM = non-rapid eye movement; AHI = apnea hypopnea index; TNF = tumor necrosis factor; IL = interleukin; DG = dentate gyrus; ROS = reactive oxygen species; NO = nitric oxide; CRP = C-reactive protein; NLR = neutrophil-to-lymphocyte ratio; PD = Parkinson’s disease; CNS = central nervous system; BBB = blood–brain barrier; LBP = *Lycium barbarum* polysaccharides; BDNF = brain-derived neurotrophic factor; TLRs = Toll-like receptors; IκB = NF-κB-inhibitor; IKK = IkB kinase; LPS = lipopolysaccharide; DAMPs = damage-associated molecular patterns; PAMPs = pathogen-associated molecular patterns; PRRs = pattern recognition receptors; MAPK = mitogen-activated; CBP = CREB binding protein; PHDs = prolyl hydroxylases; DC = dendritic cell; PKC = protein kinase C; mTOR = mammalian target of rapamycin; CAMK = Ca^2+^/calmodulin kinase; GLUT = glucose transporter; PEEP = positive end-expiratory pressure; AD = Alzheimer’s disease; EDS = excessive daytime sleepiness; SDB = sleep-disordered breathing; SNc = substantia nigra pars compacta; DA = dopaminergic.
